# Is progress being made on Canada’s palliative care framework and action plan? A survey of stakeholder perspectives

**DOI:** 10.1186/s12904-022-01074-4

**Published:** 2022-10-14

**Authors:** Barbara Pesut, Sally Thorne, Anne Huisken, David Kenneth Wright, Kenneth Chambaere, Carol Tishelman, Sunita Ghosh

**Affiliations:** 1 Professor School of Nursing, Principal Research Chair in Palliative and End-of-Life Care, University of British Columbia Okanagan. ARTS 3rdFloor, 1147 Research Road, Kelowna, BC V1V 1V7 Canada; 2 Professor School of Nursing, University of British Columbia, Vancouver, BC V6T 2B5 Canada; 3 Research Coordinator Palliative and End of Life Research Lab, University of British Columbia Okanagan, BC Kelowna, Canada; 4 Associate Professor, School of Nursing, University of Ottawa, Ottawa, ON K1H 8M5 Canada; 5 Professor Public Health, Sociology & Ethics of the End of Life, End-of-Life Care Research Group, Vrije Universiteit Brussel (VUB) & Ghent University, C. Heymanslaan 10, B-9000 Ghent, Belgium; 6 Professor of Innovative Care Karoliinska Institutet, Department of Learning, Informatics, Management and Ethics Division of Innovative Care Research, Stockholm, Sweden; 7 Associate Clinical Professor/Research Scientist, University of Alberta/Alberta Health Services, Edmonton, Canada

**Keywords:** Palliative care, Survey, Qualitative, Quality improvement, Canada

## Abstract

**Background:**

The legalization of Medical Assistance in Dying in Canada in 2016 provided new impetus for improving palliative care. This commitment to improvement included the development of a National Palliative Care Framework and Action Plan. The purpose of this study was to understand the progress made in palliative care since 2016 from the perspective of persons working and volunteering in palliative care and compare geographic differences.

**Methods:**

A digital survey was developed from goals identified in Canada’s Palliative Care Framework and Action Plan and administered online using Qualtrics. Participants were recruited through national palliative care organizations. The survey included both quantitative survey items designed to evaluate improvements across 5 domains and 29 items and included open-ended questions about impacts, innovations, and ongoing challenges. Descriptive statistics were generated for survey domains, items, and demographic variables. Geographic differences were compared using Independent-Samples Kruskal–Wallis test. Qualitative data was analyzed inductively into themes.

**Results:**

One hundred fifty surveys met inclusion criteria and were analysed. Overall, the most improvement was reported in palliative care education and the least improvement was reported in support for family caregivers. Items on which respondents reported the most improvement included healthcare provider education in palliative care, advance care planning, and use of technology. Items on which respondents reported the least improvement were respite for family caregivers, access to bereavement services, and in-home support for family caregivers. Notably, rural participants reported more statistically significant improvements in the domains of education, access, and research and data collection than their urban counterparts. However, rural participants reported less improvement in places to die when home is not preferable. The COVID-19 pandemic was a significant contributor to these perceived improvements and ongoing challenges.

**Conclusion:**

Canada’s Framework and Action Plan sets out a roadmap for improving palliative care in Canada. Participants in this survey noted significant improvements in key areas, a notable accomplishment amidst the effects of the COVID-19 pandemic. Some improvements were a result of greater use of distance technology. Further leveraging these improvements will make an important contribution to solving some of the rural and remote palliative care issues that have arisen from Canada’s unique geography.

## Background

A growing body of international agencies, such as The World Health Organization (WHO), recognize the importance of palliative care. The WHO urges its member states to strengthen and integrate palliative care into health systems, set targets, and monitor progress [1]. Despite these ambitions, universal access to palliative care is lacking. The Global Atlas of Palliative Care estimates that in 2017 over 52 million adults worldwide needed palliative care, while only 7 million received such care [2. The development of palliative care within a country is closely correlated to economic wealth and development; most palliative care is concentrated in high-income western countries [2–4].

The WHO has developed global targets for integrating palliative care into health care systems. The highest ambition is that palliative care is at the point of advanced integration [5]. From the perspective of the WHO, advanced integration is when the following indicators have been met:

- ∙ Palliative care is available from multiple service providers.
- ∙ Healthcare professionals, communities, and the public are aware of palliative services.
- ∙ Those requiring palliative care have unrestricted access to adequate pain and symptom management.
- ∙ Policies, guidelines, strategic plans, and national palliative care associations address palliative care.
- ∙ Research partnerships exist between practice and academic centers to monitor integration progress.

Canada is one of those high-income countries that, according to the WHO, has achieved advanced integration of palliative care [4, 5]. In the Canadian context, numerous organizations and individuals are involved in the delivery of palliative care including family caregivers, community and volunteer services, primary and specialized health care providers, and provincial and territorial health care systems. Service integration is widely recognized as best practice, especially across healthcare settings [6]. Qureshi et al. studied the integration of palliative care services across six levels of the Canadian health care system and found that most service integration occurred as formalized relationships at the local level, but there was less evidence of integration at the regional, provincial, or national level [7]. This occurs, in part, because healthcare services in Canada are governed and delivered at provincial and territorial levels, and so the provision of palliative services differs across provinces and territories [8].

Despite Canada’s recognition of having achieved advanced integration, Canada does not score particularly well on global rankings of palliative care. For example, whereas Canada ranked 11^th^ according to the 2015 Quality of Death Index [9], when a different set of indicators was applied on a more recent global comparison of 81 countries, Canada’s ranking fell to 22^nd^ [10]. A contributing factor to this ranking is Canada’s vast rural and remote geography; what is available in one region may not be available in another, leading to a patchwork of services and variable service delivery [11, 12]. Most specialized palliative care services are concentrated in major urban centers, while people living in rural and remote regions of the country areas often travel great distances to access care. Thus, the provision of palliative care in these smaller communities often comes down to the dedication and resourcefulness of paid providers, families, and volunteers whose personal investments are essential to high quality palliative care [13].

There are additional reasons for Canada’s relatively low rating on global indices. Canada has no formal and enforced standards for the delivery of high quality palliative care [12]. Although home care and community care are acknowledged as cost-effective sites of palliative care, and are also Canadians’ preferable places to receive care [14], most deaths occur in hospitals [11, 14]. Data from provinces that measure publicly-funded palliative home care indicate that, in 2016–2017, only 1 in 6 Canadians who died received palliative home care [15]. This lack of palliative care access at home is important because Canadians who have access to home-based palliative care are 2.5 times more likely to die at home [15, 16]. Further, there is data to suggest that primary care physicians in Canada feel less prepared to provide palliative care than primary care physicians in other countries [12]. Overall, despite numerous reports published over a 20 year period outlining ways to improve palliative care in Canada, there has been patchy uptake of those recommendations [17].

Family caregivers and community organizations contribute significantly to palliative care provision in Canada. A 2017 report prepared by the Canadian Hospice and Palliative Care Association indicated that 2.7 million Canadians 45 years and older are family caregivers to older adults living with a life-limiting illness. In addition to providing social, spiritual, and psychological care, caregivers also provided personal and medical care, care coordination and acted as patient advocates. Together, the hours of unpaid care contributed to an economic value of $25 to 26 billion CAD annually [18]. Hospice organizations in Canada contribute significantly to public education, advocacy, and direct support of patients and family. However, as most of these organizations are dependent upon donations, much of their capacity is dedicated to raising funds for the work that they do.

The legalization of Medical Assistance in Dying in 2016 provided new impetus for the further development of palliative care in Canada. At the time of legalization, the Canadian federal government committed to examining palliative care within five years and working with Canadian provinces and territories to improve access to palliative care [16]. A similar concern about the relationship between MAID and palliative care has arisen in other international contexts. For example, in 2002 in Belgium a law affirming the right to palliative care was passed at the same time as the law decriminalizing euthanasia [19]. A subsequent study exploring the development of palliative care in Belgium and the Benelux context suggested that the introduction of euthanasia supports expansion of palliative care [20]. Likewise, an Australian report that reviewed the evidence on the impact of assisted death on palliative care suggested that there is no evidence to suggest that assisted death hindered the development of palliative care [21].

In December 2017, the Canadian Federal government passed a private member’s Bill (C-277) to develop a framework for palliative care in Canada that would address gaps in access and quality of care across Canada [16, 22]. This Palliative Care Framework that was published in 2018 contains specific short and long term goals to improve palliative care in four priority areas: palliative care training and education for healthcare providers and other caregivers; measures to support palliative care providers and caregivers; research and the collection of data on palliative care; and equitable access to palliative care across Canada [16]. To further address issues uncovered during the consultation of the framework, Health Canada developed an Action Plan for Palliative Care published in 2019 [23]. The plan outlines five specific goals:

- 1. Raise awareness and understanding of how advance care planning and palliative care can improve quality of life until the end of life;
- 2. Support health system quality by improving palliative care skills and support for health care providers, families, caregivers, and communities;
- 3. Support health system quality improvement through enhanced data collection and research;
- 4. Foster improved access to palliative care for underserved populations;
- 5. Improve access to culturally sensitive palliative care for Indigenous communities.

As part of a larger program of research in which we are seeking to understand the evolution of strategies to relieve suffering at end of life in Canada, we were interested in palliative care stakeholders’ perceptions of the progress made in palliative care since 2016 in relation to the Canadian Palliative Care Framework and Action Plan. Therefore, the purpose of this study was to understand the progress made in palliative care from the perspective of persons working and volunteering in palliative care and compare geographic differences.

## Methods

### Design

A digital survey was used to gather perceptions of improvements in palliative care. The survey included both closed and open-ended survey questions. The study received ethical approval from the Behavioural Research Ethics Board at the University of British Columbia (H21–02,128).

### Data and measures

The survey instrument was developed by two members of the research team (BP, AH) from domains and items derived from the Framework on Palliative Care [16] and the Action Plan on Palliative Care [23]. The survey was developed online using Qualtrics, tested by palliative care experts, and revised accordingly. The five domains were education and training, family caregivers, community capacity, access, and research and data collection. For each item, respondents were asked the following question “In your opinion, how has [item] changed (if at all) in your geographic area since 2016?” Possible responses ranged from “Much worse” to “No change” to “Much improved” on a 5-point Likert scale, with the additional possibility for respondents to indicate if they were “Not sure.” At the end of the items for each domain, there were two open-ended questions “What in your opinion has contributed to these changes and what has been the impact of these changes?” and “Please describe any innovations/improvements in [framework dimension] in your area.” Demographics included the respondent’s professional background, primary role in palliative care, specialized education in palliative care, years of work experience, the province/territory they work/volunteer in, and the geographic area they served.

### Sample and recruitment

The target sample was healthcare providers and volunteers involved in palliative care in Canada. Demographic data was used to include only those respondents who were currently working/volunteering within palliative care. Surveys were excluded from analysis if the demographic form was not filled out. Recruitment occurred through the Canadian Hospice and Palliative Care Association (CHPCA) and the Canadian Palliative Care Nursing Association using advertisements, email lists, and posts on social media platforms (Twitter, Facebook, and Instagram). A survey link allowed participants to respond to the survey anonymously. Security settings were enabled in Qualtrics to manage duplicate responses, bots and screen for problematic responses. Information about the survey was also fanned out through memberships lists of various Provincial/Territorial Canadian palliative care networks. The survey was active for two months, from mid-October to mid-December 2021. Given the nature of a digital survey, respondents self-selected to be part of the study. Respondents were eligible to complete the survey when they received the link and consented online to participate in the study. Surveys in which respondents completed the consent, the demographic form, and at least two questions of the survey were included for analysis. As the demographic questions were completed last this meant that most surveys included in the analysis were complete.

### Quantitative analysis

Statistical data analyses were conducted in SPSS V.26. Descriptive statistics (frequencies, percentages, median/inter quartile range (IQR) and means/standard deviations) were generated for survey domains, items, and the socio-demographic variables. Missing cases, and “not sure” responses were excluded from analysis.

#### Overall perceptions of improvement by domain

As Cronbach’s alpha showed good reliability within each domain (0.75–0.90), analysis of each domain and comparison between domains was done to better understand the overall progress. Means and standard deviations as well as the median (IQR) were calculated for each domain. The total possible score was calculated by multiplying the number of items in the domain by 5 to reflect the best possible score on the Likert scale (e.g. education = five items × 5 Likert options per items = 25 points). Only participants who responded to all items within a particular domain were included in this analysis. Mean domain scores were then compared across geographic areas and urban/rural context using independent Kruskal–Wallis tests.

#### Comparisons of Geographic regions and urban and rural areas by items

As the data did not meet assumptions of normality, Independent-Samples Kruskal–Wallis tests were used to compare geographic differences across Canada. For the univariate comparison across the geographical regions a *p*-value < 0.10 level was used, as the traditional level of *p*-value < 0.05 level may fail to identify important factors [24]. For purposes of comparing geographic regions of Canada, provinces and territories were collapsed into four areas “West” (British Columbia), “Prairies” (Alberta, Saskatchewan, Manitoba), “Central” (Ontario) and “Atlantic” (Nova Scotia, New Brunswick, Prince Edward Island, Newfoundland). Due to small sample sizes, (*n* = 4) respondents living in the Territories and Quebec were excluded from the analysis. For purposes of comparing urban and rural responses, the response options of urban, small urban, rural, or remote areas were collapsed into two groups “Urban” (Urban/ Small Urban) and “Rural” (Rural and Remote). Participants who indicated they worked in both urban and rural areas (*n* = 14) were excluded from the analysis.

### Qualitative analysis

Qualitative data from the open-ended survey responses was coded in NVivo 12 Pro. Data under each domain was analyzed inductively by first coding into open codes and then grouping together according to content-based themes. To support analytic rigor, two members of the research team constructed and negotiated the codes.

## Results

A total of 150 valid responses were included in the data analysis. Sample sizes for each domain and item varied because there was an option for respondents to indicate “not sure”. The education domain had the most engagement (average n across items = 135) and the research and data collection domain had the least engagement (average n across items = 92.5).

Table 1 provides an overview of the respondents based on their demographic characteristics. The majority of the 150 participants were health care professionals (54.6%), and 42% of those were nurses. About one-fourth of participants represented perspectives from communities and hospices, including people in leadership positions, hospice employees, administrative roles, volunteers, and spiritual care. Of the healthcare providers, 40.6% were working as specialized palliative care providers with a further 11.2% working as a primary care (non-specialized) palliative provider. The majority of respondents were from Ontario (24.7%) and British Columbia (22%) with 61% working in large urban or small urban areas. Respondents had a depth of experience in palliative care with almost half the sample reporting more than 10 years of experience. Further, many participants were nurses who are well-positioned to speak to changes in palliative care because of their role within the healthcare system.

**Table 1:** Demographic characteristics of survey respondents (*n* = 150)

Professional Background	n (%)
Nurse	59 (39.3)
Other (e.g., leadership positions, hospice employees, administrative roles, volunteers with professional background other than listed)	36 (24)
No professional background	11 (7.3)
Social Worker	9 (6)
Physician	8(5.3)
Nurse Practitioner	4 (2.7)
Occupational Therapist	2(1.3)
Pharmacist	0 (0)
Missing	21(14)
**Primary role in palliative care**	
Specialized palliative care provider (adult population)	58 (40.6)
Volunteer	26 (18.2)
Other (e.g., educators, executive directors, volunteer coordinators, spiritual care)	24 (16.8)
Non-specialized palliative care provider (e.g., community health nurse, family physician)	16 (11.2)
System level decision-maker/leader for palliative care or palliative approach to care	14 (9.8)
Researcher	5 (3.5)
Specialized palliative care provider (pediatric population)	0 (0)
Missing	7 (4.7)
**Do you have education in specialized palliative care or in a palliative approach to care?**	
Yes	99 (66)
No	23 (15.3)
Unsure	8 (5.3)
Missing	20 (13.3)
**# years working/volunteering in palliative care**	
1–4	25 (16.7)
5–9	29 (19.3)
10–14	27 (18)
15–19	13 (8.7)
> 20	33 (22)
Missing	23 (15.3)
**Province/ Territory work in**	
British Columbia	33 (22)
Alberta	14 (9.3)
Saskatchewan	7 (4.7)
Manitoba	4 (2.7)
Ontario	37 (24.7)
Quebec	3 (2)
Prince Edward Island	6 (4)
Nova Scotia	9 (6)
New Brunswick	10 (6.7)
Newfoundland/Labrador	4 (2.7)
Northwest Territories	1 (0.7)
Nunavut	0 (0)
Work in more than one province	3 (2)
Yukon	0 (0)
Missing	19 (12.7)
**Geographic area (one or more answers possible)**	
Urban (> 100,000)	63 (37)
Small urban (10,000 to 99,000 population)	50 (30)
Rural (< 10,000 population) Remote	33 (19) 2 (1)
Missing	21(12)

### Survey domain analysis

Statistical analysis indicated modest improvements within each of the five framework domains (Table 2). Perceived improvements within each domain were the lowest for the family caregiver domain and the highest for the palliative education domain.

**Table 2:** Overview of perceived progress within each survey domain

	Education	Family Caregiver	Community Capacity	Access	Research and Data Collection
Mean domain score (SD)	16.9 (3.3)	18.5 (4.3)	13.4 (2.2)	32.6 (4.8)	12.9 (2.5)
Total possible domain score	25	30	20	50	20
N	104	110	109	73	82

Rural participants reported statistically significant improvements in the domains of education, access, and research and data collection relative to their urban counterparts (Table 3).

**Table 3:** Differences on mean domain scores across urban/rural areas of Canada

	Urban Mean (SD) Median (IQR)	Rural Mean (SD) Median (IQR)	*p*-value
Education	16.48 (3.14) 16.0 (15.0–18.0)	18.38 (2.84) 18.5 (16.3–19.3)	0.039 *
Family Caregiver	17.82 (4.28) 18.00 (14.3 -20.8)	19.55 (3.66) 21.5 (18.8 -24.3)	0.095*
Community Capacity	13.13 (2.29) 13.0 (12.0 -14.0)	13.83 (1.95) 14.5 (14.0 -15.0)	0.278
Access	31.75 (5.02) 31.5 (28.3 -34.0)	34.69 (3.17) 37.0 (34.3 -37.8)	0.021*
Research and Data Collection	12.33 (2.35) 12.0 (12.0 -13.8)	14.08 (1.93) 15.5 (13.5 -16.0)	0.023*

^*^Significance *p* < 0 .10

There were no statistically significant differences on domain scores across the geographic regions of Canada (Table 4).

**Table 4:** Differences on domain scores across aggregated geographic areas of Canada

	BC Mean (SD) Median (IQR)	Prairies Mean (SD) Median (IQR)	Ontario Mean (SD) Median (IQR)	Maritimes Mean (SD) Median (IQR)	*p*-value
Education	17.17 (4.15) 19.5 (17.8 -20.8)	16.63 (2.92) 19.5 (17.5 -20.0)	17.13 (2.71) 16.0 (14.0 -18.0)	16.62 (3.38) 16.0 (14.8 -17.3)	0.808
Family Caregiver	17.88 (3.55) 20.5 (18.3 -22.0)	18.84 (4.10) 20.0 (19.0 -24.0)	18.00 (4.24) 16.0 (12.0–18.5)	18.64 (4.49) 18.5 (13.8–21.0)	0.916
Community Capacity	13.55 (2.87) 14.5 (13.3 -15.8)	13.00 (1.86) 15.0 (12.3–15.0)	12.88 (1.52) 13.0 (12.0–13.0)	13.71 (2.63) 13.0 (12.0–14.0)	0.345
Access	32.83 (5.80) 37.0 (34.0–38.5)	32.57 (4.16) 35.0 (31.5 -39.3)	32.39 (4.39) 32.0 (29.5–34.5)	32.44 (5.67) 31.5 (27.5 -34.0)	0.918
Research and Data Collection	13.63 (2.92) 15.0 (12.5–165.0)	13.17 (2.23) 14.5 (12.5–15.8)	12.35 (2.2.8) 12.0 (12.0–14.0)	12.58 (2.55) 12.5 (12.0 – 13.0)	0.553

^*^Significance *p* < 0 .10

### Survey item analysis

**Fig. 1:**
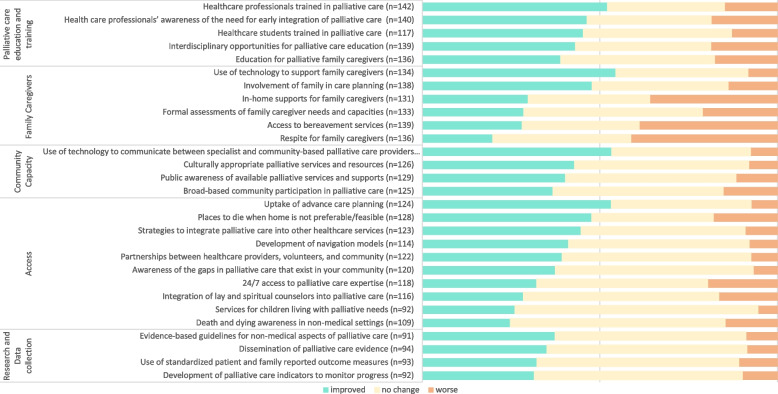
Respondents’ perceptions of the development of the Palliative Care Framework Measures since 2016

A graph of respondents’ perceptions of changes on the survey items are shown in Fig. 1. Items that indicated improvement as perceived by the majority of respondents were: use of technology to support family caregivers (54.5%), use of technology to communicate between specialists and community-based palliative care providers (53.3%), uptake of advance care planning (53.2%), and health care professionals trained in palliative care (52.1%). The three items that were perceived by the most respondents to have worsened were: respite for family caregivers (41.2%), access to bereavement services (38.8%), and in-home support for family caregivers (35.9%).

Table 5 reports the mean scores and medians for each survey item by geographic distribution. Mean scores ranged from 2.5 to 3.7 on a 5-point Likert scale. Items that received a mean score of less than 3 were: in home support for family caregivers (West, Central regions), respite for family caregivers (all regions), integration of lay and spiritual counsellors into palliative care (West), and use of standardized patient and family reported outcome measures (Central). There were statistically significant differences in the use of standardized patient and family reported outcome measures, with the central region reporting lower scores in this area than in other regions and in the integration of lay and spiritual counselors into palliative care, with British Columbia reporting lower scores than in other regions.

**Table 5:** Differences on survey items by aggregated geographic areas of Canada (5-point Likert Scale)

		**West**		**Prairies**		**Ontario**		**Maritimes**		
		**N**	**M (SD) Median(IQR)**	**N**	**M (SD) Median(IQR)**	**N**	**M (SD) Median(IQR)**	**N**	**M (SD) Median(IQR)**	***p*-value**
**Education Measures**	Healthcare professionals trained in palliative care	32	3.2(1.2) 4.0 (3.0 -4.8)	23	3.6 (.7) 4.0 (3.3 -4.8)	37	3.5 (.8) 4.0 (3.0–4.0)	27	3.6 (.8) 4.0 (3.0–4.0)	0.397
	Healthcare students trained in palliative care	20	3.7(.9) 4.0 (4.0 -4.0)	20	3.4 (.7) 4.0 (3.3–4.0)	32	3.3 (.8) 3.0 (3.0–4.0)	23	3.2 (.8) 3.0 (3.0–3.0)	0.211
	Education for palliative family caregivers	30	3.3 (.8) 3.5 (3.0–4.0)	22	3.2 (.7) 3.5 (3.0–4.0)	33	3.4 (.8) 3.0 (3.0 -3.0)	28	3.1 (.9) 3.0 (2.8–3.0)	0.391
	Health care professionals’ awareness of the need for early integration of palliative care	31	3.2 (1.1) 4.0 (3.3–4.8)	23	3.4 (.7) 4.0 (4.0–4.0)	36	3.5 (.3) 3.0 (3.0–4.0)	27	3.3 (.7) 3.0 (3.0–4.0)	0.497
	Interdisciplinary opportunities for palliative care education	30	3.1 (1) 3.5 (3.0–4.0)	24	3.2 (.8) 4.0 (3.3–4.0)	36	3.5 (.3) 3.0 (3.0–4.0)	26	3.2 (.9) 3.0 (2.8–4.0)	0.383
**Family Caregiver measures**	Formal assessments of family caregiver needs and capacities	27	3.0 (.8) 3.5 (3.0–4.0)	21	3.2 (.6) 3.0 (3.0–3.8)	35	3.0(.9) 3.0 (2.0–3.0)	27	3.0 (.9) 3.0 (2.0–3.3)	0.864
	Involvement of family in care planning	31	3.2 (.9) 3.0 (3.0–3.8)	23	3.6 (.7) 3.5 (3.0–4.8)	37	3.4(.8) 3.0 (2.0–4.0)	25	3.4 (.8) 3.0 (2.8–4.0)	0.392
	Use of technology to support family caregivers	27	3.5 (.9) 4.0 (3.0–4.8)	24	3.4(.6) 4.0 (3.3–4.0)	36	3.5 (.9) 3.0 (3.0–4.0)	25	3.6 (.6) 3.5 (3.0–4.0)	0.569
	In-home supports for family caregivers	26	2.9 (1) 3.0 (2.3–4.0)	20	3.1 (1.2) 3.5 (3.0–4.8)	36	2.6 (1.1) 2.0 (1.0–3.0)	25	3 (1) 3.0 (2.0–3.0)	0.488
	Respite for family caregivers	28	2.6 (.9) 3.0 (2.3 -3.8)	22	2.7 (.9) 3.0 (3.0–3.8)	36	2.5(1) 2.0 (1.0–3.0)	27	2.9 (.9) 3.0 (2.0–3.3)	.0461
	Access to Bereavement Services	30	3.1 (.9) 3.0 (3.0–3.8)	23	3.1 (1) 3.0 (3.0 -3.8)	36	3.1(1.2) 2.0 (1.5–4.0)	28	3.0 (1) 3.0 (2.0–4.0)	0.943
**Community Capacity Measures**	Broad-based community participation in palliative care	27	3.2 (.9) 3.0 (3.0 -3.8)	24	3.2 (.8) 3.0 (3.0–3.8)	35	3.1 (.7) 3.0 (3.0–3.0)	25	3.3 (.9) 3.0 (3.0–4.00)	0.758
	Culturally appropriate palliative services and resources	28	3.5 (.8) 4.0 (3.3–4.0)	24	3.3 (.8) 3.5 (3.-4.0)	35	3.3 (.5) 3.0 (3.0–3.5)	26	3.3(.8) 3.0 (3.0–4.0)	0.419
	Public awareness of available palliative services and supports	30	3.3 (1) 4.0 (3.0–4.0)	23	3.4 (.7) 4.0 (3.3–4.0)	36	3.3 (.6) 3.0 (3.0–4.0)	26	3.4 (.7) 3.0 (3.0–4.0)	0.852
	Use of technology to communicate between specialist and community-based palliative care providers	25	3.6 (.9) 4.0 (3.0–4.8)	20	3.3 (.6) 4.0 (3.3–4.0)	35	3.5 (.8) 3.0 (3.0–4.0)	25	3.6 (.7) 3.0 (3.0–3.0)	0.328
**Access Measures**	Development of navigation models	26	3.3 (.9) 4.0 (3.0–4.0)	21	3.4 (.5) 3.5 (3.0–4.0)	32	3.4 (.7) 3.0 (3.0–3.5)	25	3.2 (.6) 3.0 (2.8–3.0)	0.529
	24/7 access to palliative care expertise	27	3.0 (1) 4.0 (3.3–4.0)	22	3.2 (.7) 3.0 (3.0–3.8)	33	3.1 (.9) 3.0 (2.0–3.0)	26	3.3 (.8) 3.0 (2.0–4.0)	0.781
	Death and dying awareness in non-medical settings	26	3.1 (.7) 3.0 (3.0–4.0)	21	3.2 (.6) 3.5 (3.0–4.0)	33	3.2 (.6) 3.0 (2.0–3.0)	20	3 (.8) 3.0 (2.0–3.0)	0.467
	Uptake of advance care planning	29	3.7 (.7) 3.5 (3.0–4.0)	23	3.5(.7) 4.0 (3.3–4.0)	37	3.6 (.7) 3.0 (3.0–4.0)	25	3.2 (1) 3.0 (2.0–3.3)	0.270
	Strategies to integrate palliative care into other healthcare services	28	3.4 (1) 4.0 (3.3 -4.0)	24	3.3 (.8) 3.5 (3.0–4.0)	36	3.4 (.6) 3.0 (3.0–4.0)	25	3.4 (.7) 3.0 (3.0–4.0)	0.841
	Partnerships between healthcare providers, volunteers, and community	28	3.2 (.7) 3.0 (3.0–3.8)	23	3.4 (.9) 3.5 (3.0–4.8)	35	3.5 (.7) 3.0 (3.0–4.0)	26	3.5 (.6) 3.0 (3.0–4.0)	0.392
	Integration of lay and spiritual counselors into palliative care	27	2.8 (.8) 3.0 (3.0–3.0)	22	3.0 (.8) 3.5 (3.0–4.0)	32	3.3 (.8) 3.0 (3.0–4.0)	25	3.2 (.9) 3.0 (2.8–4.0)	0.067*
	Places to die when home is not preferable/feasible	29	3.2 (1) 4.0 (4.0–4.8)	23	3.0 (.9) 3.5 (3.0–4.0)	37	3.4 (1) 4.0 (3.0–4.0)	28	3.6 (.8) 4.0 (3.0–4.0)	0.124
	Services for children living with palliative needs	16	3.1 (.5) 3.0 (3.0–3.8)	19	3.3 (.6) 3.0 (3.0–3.8)	28	3.2 (.6) 3.0 (3.0–3.5)	22	3.3 (.8) 3.0 (3.0–3.0)	0.879
	Awareness of the gaps in palliative care that exist in your community	28	3.3 (.8) 4.0 (3.0–4.0)	23	3.6 (.7) 4.0 (3.3 -4.0)	33	3.4 (.7) 3.0 (3.0–4.0)	25	3.3 (.6) 3.0 (3.0–3.3)	0.515
**Research and Data Collection**	Evidence-based guidelines for non-medical aspects of palliative care	17	3.4 (.8) 4.0(3.0–4.0)	19	3.3 (.6) 4.0 (3.3–4.0)	28	3.3 (.8) 3.0 (3.0–4.0)	22	3.1 (.7) 3.0 (3.0–3.0)	0.568
	Dissemination of palliative care evidence	19	3.4 (.8) 4.0 (3.3–4.0)	20	3.4 (.6) 4.0 (3.3–4.0)	27	3.2 (.6) 3.0 (3.0–3.5)	22	3.1 (.8) 3.0 (3.0–3.3))	0.611
	Use of standardized patient and family reported outcome measures	19	3.5 (.7) 4.0 (3.0–4.0)	19	3.3 (.6) 3.5 (3.0–4.0)	27	2.9 (.7) 3.0 (2.0–3.0)	22	3.3 (.7) 3.0 (3.0–4.0)	0.056*
	Development of palliative care indicators to monitor progress	18	3.3 (.7) 3.5 (3.0–4.0)	19	3.2 (.6) 3.0 (3.0–3.8)	30	3.1 (.7) 3.0 (3.0–3.5)	21	3.3 (.7) 3.0 (3.0–4.0)	0.513

^*^Significance *p* < 0.10

Table 6 reports the mean scores and median for each item by rural versus urban location. Mean scores ranged from 2.6 to 3.7 on a 5-point scale. Items that received a mean score of less than 3 were: formal assessments of family caregiver needs and capacities (urban), in-home supports for family caregivers (urban and rural) and respite for family caregivers (urban and rural). Statistically significant differences between urban and rural areas were reported in healthcare students trained in palliative care, involvement of family in care planning, public awareness of available palliative services and supports, places to die when home is not preferable, evidence-based guidelines for non-medical aspects of palliative care, and the dissemination of palliative care evidence. Rural participants reported better improvement on all these indicators except for places to die when home is not preferable.

**Table 6:** Differences on survey items by aggregated urban and rural areas (5-point Likert Scale)

		Urban		Rural		
		N	M (SD) Median (IQR)	N	M (SD) Median (IQR)	p-value
Education Measures	Healthcare professionals trained in palliative care	91	3.4 (.9) 4.0 (3.0–4.0)	22	3.6 (.8) 3.5 (3.0–4.3)	0.279
	Healthcare students trained in palliative care	76	3.3 (.8) 3.0 (3.0–4.0)	14	3.6 (.7) 3.5 (2.8–4.0)	0.093*
	Education for palliative family caregivers	85	3.2 (.8) 3.0 (3.0–3.0)	22	3.4 (.7) 3.5 (3.0–4.0)	0.332
	Health care professionals' awareness of the need for early integration of palliative care	89	3.3 (.8) 3.0 (3.0–4.0)	22	3.3 (1.0) 4.0 (2.8–4.8)	0.912
	Interdisciplinary opportunities for palliative care education	89	3.2 (.9) 3.0 (3.0–4.0)	22	3.4 (.7) 4.0 (3.0–4.0)	0.389
Family Caregiver measures	Formal assessments of family caregiver needs and capacities	81	3.0 (.8) 3.0 (3.0–4.0)	22	3.2 (.6) 3.0 (3.0–4.0)	0.162
	Involvement of family in care planning	88	3.3 (.8) 3.0 (3.0–4.0)	21	3.7 (.7) 4.0 (3.8–4.3)	0.095*
	Use of technology to support family caregivers	85	3.5 (.8) 3.0 (3.0–4.0)	20	3.6 (.5) 4.0 (4.0–4.0)	0.972
	In-home supports for family caregivers	80	2.8 (1.0) 3.0 (2.0–3.0)	22	3 (1.0) 4.0 (2.0–4.3)	0.543
	Respite for family caregivers	85	2.6 (.9) 3.0 (2.0–3.0)	22	2.7 (.9) 3.0 (2.0–4.0)	0.744
	Access to Bereavement Services	88	3.0 (1.1) 3.0 (2.0–3.0)	23	3.3 (.8) 4.0 (3.0–4.0)	0.331
Community Capacity Measures	Broad-based community participation in palliative care	84	3.1 (.8) 3.0 (3.0–4.0)	21	3.2 (.8) 3.0 (3.0–3.3)	0.969
	Culturally appropriate palliative services and resources	87	3.3 (.7) 3.0 (3.0–4.0)	21	3.4 (.8) 3.5 (3.0–4.0)	0.969
	Public awareness of available palliative services and supports	88	3.2 (.8) 3.0 (3.0–4.0)	21	3.6 (.7) 4.0 (4.0–4.0)	0.085*
	Use of technology to communicate between specialist and community-based palliative care providers	82	3.5 (.8) 3.0(3.0–4.0)	19	3.6 (.6) 4.0 (3.8–4.0)	0.458
Access Measures	Development of navigation models	82	3.3 (.7) 3.0 (3.0–4.0)	18	3.5 (.6) 4.0 (3.0–4.3)	0.272
	24/7 access to palliative care expertise	84	3.1 (.9) 3.0 (2.0–3.0)	21	3.2 (.7) 3.5 (3.0–4.0)	0.451
	Death and dying awareness in non-medical settings	77	3.1 (.7) 3.0 (3.0–3.0)	20	3.3 (.6) 3.5 (3.0–4.0)	0.218
	Uptake of advance care planning	86	3.5 (.8) 3.0 (3.0–4.0)	21	3.7 (.6) 4.0 (3.8–4.0)	0.252
	Strategies to integrate palliative care into other healthcare services	88	3.4 (.8) 3.0 (3.0–4.0)	22	3.6 (.7) 4.0 (3.0–4.0)	0.242
	Partnerships between healthcare providers, volunteers, and community	87	3.3 (.7) 3.0 (3.0–4.0)	21	3.4 (.8) 4.0 (3.0–4.3)	0.629
	Integration of lay and spiritual counselors into palliative care	85	3.0 (.9) 3.0 (3.0–4.0)	18	3.2 (.7) 4.0 (3.8–4.0)	0.368
	Places to die when home is not preferable/feasible	89	3.4 (1.0) 4.0 (3.0–4.0)	23	3.1 (.8) 3.5 (2.8–4.3)	0.091*
	Services for children living with palliative needs	64	3.1 (.6) 3.0 (3.0–3.0)	18	3.4 (.7) 3.0 (3.0–4.0)	0.139
	Awareness of the gaps in palliative care that exist in your community	83	3.3 (.7) 3.0 (3.0–4.0)	21	3.4 (.8) 4.0 (3.0–4.3)	0.877
Research and Data Collection	Evidence-based guidelines for non-medical aspects of palliative care	66	3.2 (.7) 3.0 (3.0–4.0)	13	3.5 (.5) 4.0 (3.8–4.0)	0.062*
	Dissemination of palliative care evidence	68	3.1 (.7) 3.0 (3.0–4.0)	15	3.5 (.5) 4.0 (3.8–4.0)	0.027*
	Use of standardized patient and family reported outcome measures	70	3.1 (.7) 3.0 (3.0–3.0)	13	3.4 (.5) 3.5 (3.0–4.0)	0.208
	Development of palliative care indicators to monitor progress	69	3.1 (.7) 3.0 (3.0–4.0)	14	3.4 (.5) 4.0 (3.0–4.0)	0.102

^*^Significance *p* < 0 .10

### Qualitative findings

Survey participants provided a number of qualitative comments to support their perceptions of improvements, or lack thereof, in palliative care. These will be reported under the survey domains.

#### Palliative care education and training

In the survey, more than half of respondents (52%) reported that the availability of health care professionals trained in palliative care had improved. In the open-ended responses, participants indicated that during the COVID pandemic, the shift to virtual learning allowed for more effective and timely education. Respondents cited many virtual opportunities such as Comprehensive Advanced Palliative Care Education, Pallium’s Learning Essential Approaches to Palliative Care (LEAP https://www.pallium.ca/courses) and Project ECHO (https://www.pallium.ca/palliative-care-echo-project/). In particular, the LEAP program was frequently mentioned because it offered a range of courses for different healthcare providers, settings, and medical subspecialties. This included an innovation that provided palliative care training to paramedics to treat palliative needs at home and reduce hospital admissions. Respondents also wrote about the positive impact of less formal learning opportunities for healthcare providers to be trained in palliative care, including mentorships, meetings, and workplace training/education sessions.

Respondents further described barriers to improving palliative education and training. Examples of these barriers included a failure of leadership to value and promote palliative education and heavy staff workloads that did not allow for high quality palliative care. One respondent described how, even though more staff had received training in palliative care, workplace realities prevented them from enacting such care. “The care that patients are receiving has not improved and it is often worse because of issues unrelated to education, such as time and caseloads.” The cost of education was an additional barrier. Some employers did not cover course costs, and in some cases, access was cost-prohibitive.

Respondents perceived less improvement regarding health care students trained in palliative care, with 45% indicating improvement and 42% reporting no change. In the open-ended responses, some respondents reported that educational institutions had integrated palliative care into degree curriculums, medical residency rotations, or offered elective courses. However, other respondents wrote that education institutions did not sufficiently train new graduates for careers in palliative or home care teams.

Just under half of the respondents to the survey (46%) reported that health care professionals’ awareness of the need for early integration of palliative care had improved, and 35% reported no change. In the open-ended responses, respondents wrote about ways their managers had prioritized learning about early integration by providing education and funding. In contrast, respondents commonly cited patient outcomes such as late referrals, emergency visits, or “horrible home deaths” as a consequence when healthcare providers were unfamiliar with the importance of early integration of palliative care.

In the survey, 43% of respondents perceived improvement in interdisciplinary opportunities for palliative care education, while 38% perceived that there was no change. In the open-ended responses, respondents wrote that although there are efforts to expand palliative care knowledge into other healthcare professions, it was still very focused on nurses’ and physicians’ roles. These respondents suggested that more could be done to learn from other healthcare providers like spiritual care providers and social workers.

Respondents perceived that family caregiver education had either not changed (43%) or improved (39%). In the open-ended responses, respondents described how providing education for family caregivers has largely been delegated to hospice societies and other non-profit community organizations.

#### Family caregiver support

More than half of the respondents (54.5%) perceived that the use of technology to support family caregivers had improved. Respondents wrote that the shift to virtual care during the pandemic resulted in better access for family/caregivers and better communication between care teams, family/caregivers, and patients (e.g. virtual medical visits). However, such virtual care was perceived negatively if it was prolonged beyond the time when in-person visits were again feasible. Also, virtual visits tended to render invisible the challenges that families were struggling with. “The increase in virtual support has been helpful in some ways. However, it has also led to patients being supported virtually longer than they would have been supported as an outpatient and the transition to in-home care is delayed.” Respondents perceived improvement (48%) or no change (38%) involving the family in care planning. Respondents wrote about the positive impact of advance care planning education and recent campaigns emphasizing the importance of family and their support in advance care planning initiatives.

Despite these gains, only up to 30% of respondents perceived that the following items had improved: in-home support for family caregivers (30%), formal assessments for family/caregiver’s needs (29%), access to bereavement services (28%) and respite for family caregivers (20%). In the open-ended responses, participants wrote about the range of services lacking but needed to address the needs of family caregivers under these items. These services included healthcare navigation, after-hours care, family visitation, counselling, and grief and bereavement support. Respondents perceived that family members should receive more and earlier help in dealing with their grief and bereavement. Where available, volunteers were said to fill an important role in grief and bereavement support to family caregivers. Moreover, respondents described how increased privatization of home services led to higher costs for families, and 24/7 access to help for caregivers was not easily accessible. These issues were exacerbated by ongoing staff shortages in community and clinical care teams. Some of these shortages were related to austerity measures and low pay for home support workers. As a result, family caregivers shouldered more of the caregiving responsibilities.

#### Primary care and community capacity

Survey respondents indicated that the use of technology between specialist and community-based palliative care providers had improved (53%) or not changed (39%). In the open-ended responses, respondents wrote about the impact of COVID and the subsequent increased use of technology. For example, care teams used video-calling platforms for meetings and appointments. Registered Nurses and physicians provided email, text, and phone support to community care teams. One respondent mentioned their community had a virtual e-dashboard to share documentation from paramedic visits with community teams.

Most respondents perceived no change (49%) or improvement (43%) in culturally appropriate palliative services and resources. In the open-ended responses, several respondents perceived that “the provision of palliative care follows a middle-class norm” and that safe care was lacking for Indigenous, transgender, structurally vulnerable, and immigrant and refugee populations. Respondents wrote that increased funding to support the development of culturally appropriate services and training opportunities for health care providers had positive impacts when it was available. They provided examples of courses on Indigenous cultural safety and other intersectionalities, such as LGBTQIA + training.

In the survey, respondents’ views varied on whether public awareness of available palliative services and supports had not changed (48%) or improved (40%). Some respondents wrote of the consequences of living in a death-denying society while others acknowledged the number of organizations working to cultivate a death embracing society through innovative community outreach programs. One respondent working within hospice wrote of the challenges of relying primarily on volunteer organizations to raise public awareness. “It puts more pressure on volunteer community-based organizations to do the work and there are no funds for that. Not to mention a lack of understanding within the medical system and a hesitation to inform their patients/clients about palliative care.”

#### Access

Overall, respondents perceived that the uptake of advance care planning had improved (53%). Further, 48% indicated that places to die when home is not preferable had improved, 45% indicated that strategies to integrate palliative care into other healthcare services (e.g., long term care) had improved, and 41% indicated that the development of navigation models had improved. The availability of hospice beds in the community was cited as an important factor in whether there had been an improvement related to places to die. Respondents further wrote of the importance of leadership and specific service delivery models when considering palliative integration into long term care and medical units.

Only 39% of respondents indicated that partnerships between healthcare providers, volunteers, and the community had improved. In the open-ended responses, respondents described innovations such as regional palliative care working groups, community partnerships, integrating an interdisciplinary approach to care, and adopting a health authority-wide palliative approach to care to strengthen these partnerships. Further, only 32% reported improvement in 24/7 access to palliative care expertise. In the open-ended responses, respondents commented that palliative care and community teams’ access was restricted to workweek hours in many jurisdictions, leaving little coverage for evenings and weekends, “People do not die in a 9–5 weekday”. Respondents living in communities where paramedics had palliative care training wrote favourably about the innovation and its success in improving 24/7 access to palliative care and reducing ER visits in their local hospitals.

Several other access issues show little sign of improvement on the survey. For example, only 29% reported improvement in the integration of lay and spiritual counselors and this area was perceived to be vulnerable to budget cuts. Only 26% perceived improvements in services for children with palliative needs, writing that most of these services were concentrated in urban areas. Finally, only 38% reported improvements in public awareness of the gaps in palliative care that exist in community and only 25% reported improvements in death and dying awareness in schools and workplaces.

#### Research and data collection

This domain had the fewest survey respondents. Those who did respond largely perceived no change (54%) in evidence-based guidelines for non-medical aspects of palliative care, no change (56%) in disseminating palliative care evidence, in the use of standardized patient and family outcome measures (57%), and in the development of palliative care indicators to monitor progress (59%). Although data in the open-ended responses were limited, respondents reported that palliative care research was often underfunded and respondents were unaware of studies or data collection initiatives at the regional or national level and within community, hospital or hospital or home care environments.

## Discussion

Respondents to this survey perceived that the most improvement in palliative care had occurred in palliative education for healthcare providers, the use of technology to connect the caregiving team, and the uptake of advance care planning. The domain that was perceived to have improved the least was related to family caregivers. Notably, respondents who worked in rural areas reported statistically significant greater improvement in the domains of education, access, and research and data collection than their urban counterparts. The COVID-19 pandemic was cited as a significant contributor to these perceived gains and challenges. Few statistically significant differences were reported across provincial geographic areas. Overall, respondents to this survey indicated that over the past five years palliative care in Canada had largely improved or stayed the same. This is a significant achievement in consideration of the strains put on the healthcare system by the pandemic.

Some of the strongest perceived gains were in the area of palliative care education for healthcare providers. Participants wrote of the increased availability of standardized, multi-disciplinary education that was available online. Pallium Canada was mentioned frequently as an important source of education. Pallium is a Canadian non-profit organization whose major goal is to build palliative care capacity at the level of primary care. Their Learning Essential Approaches in Palliative Care (LEAP) education is well-developed with multiple modules targeted at various professions and settings [25]. However, participants also indicated that education in and of itself could not improve palliative care. Educational gains could only be realized by also improving the social, organizational, political, and economic contexts in which palliative care is provided [26, 27]. Factors such as leadership, human resource challenges, system coordination, and workload influence how and whether palliative knowledge and principles can be applied.

The wide-spread use of technology was also perceived to have improved significantly, largely because of the COVID-19 pandemic [28]. A recent report on the impact of COVID-19 on home and community-based palliative care in Canada provides insight into these improvements [29]. The use of technology resulted in better access for rural and remote populations, enhanced ability for palliative providers to conduct more visits in a day, and hence, more timely access for patients. However, challenges with using technology included poor connectivity in rural and remote settings and problems with assessing the home environment and non-verbal patient and family cues in a virtual visit. In their analysis of the risks and benefits of in-person versus virtual visits for palliative care, Hawkins et al., [30] suggested that it will be important to decide on the role of technology in palliative care when going forward in a post-COVID world. Further research is required to better understand how to use technology to support a compassionate, person-centered palliative care approach.

Advance care planning became particularly important during the COVID-19 pandemic [31, 32]. Whereas there had been a major focus on advance care planning in Canada prior to the pandemic, including an extensive public campaign and a national advance care planning day [33], the realities of COVID-19 may have provided greater impetus for these discussions. Persons living with a palliative diagnosis were reluctant to use institutional healthcare because of a perceived risk of infection and because visiting policies were so restrictive. Therefore, discussions about the goals of care had to occur before the possibility of becoming hospitalized arose. There is an urgent need to build upon the new and innovative approaches to advance care planning that were driven by the COVID-19 public health emergency [29].

Despite improvements in education, technology and advance care planning, survey participants perceived that many aspects of the support received by family caregivers had worsened. This included respite, in-home supports, and access to bereavement services. Challenges experienced by palliative family caregivers have been well documented for decades. These include taking on primary responsibility for care without that role necessarily being respected by healthcare providers, challenges navigating a complex system, and physical and emotional effects from heavy caregiving responsibilities [34–37]. These challenges were exacerbated by the COVID-19 pandemic. With less in-home support from professionals and volunteers, and a fear of admission to hospital, family caregivers were required to fill in the gaps resulting in an increase in burnout and compassion fatigue [29]. Further, there is a robust body of literature documenting the potential negative physical and mental impacts of family caregiving during the palliative and bereavement phases [35], particularly when there is poor social support, family conflict, or mental health difficulties to begin with [38]. However, there are also many evidence-based treatments and guidelines specific to supporting family caregivers. For example, Australian guidelines provide 14 principles and 20 specific guidelines for the support of family caregivers of palliative patients [39].

Finally, survey participants indicated that there is room for improvement in enhancing the capacity of important palliative team members such as spiritual care providers and volunteers, a finding that has been echoed in other studies [40]. This emphasis on including community-based team members has become more prevalent in the palliative literature. In reflecting on the palliative care framework itself, Gallagher and Marshall [41] highlight the importance of looking beyond access to care issues (i.e., formal healthcare) toward building capacity in the community and public. This is often referred to as a compassionate, public-health approach to palliative care [37, 42, 43]. This approach to care has further been championed by the Canadian Society for Palliative Care Physicians, something they refer to as a bottom-up approach to palliative care [17]. Volunteer hospice societies make vital contributions to palliative care in Canada and yet experience numerous barriers to doing their work well [44]. The valuable work of these volunteers was severely limited during the COVID-19 pandemic [45]. There is an urgent need to take practical steps, such as enhanced funding, to better support those organizations that contribute to building community capacity.

The finding that most elements of palliative care had improved more in rural communities than in their urban counterparts is a particularly compelling finding in relation to Canada’s unique geography and the challenges inherent in providing palliative care to rural and remote communities. Such improvements in rural and remote care are necessary to ensure that everyone in Canada has access to high quality palliative care, not just those living in urban centers. However, one indicator, places to die when home is not preferable/feasible, particularly bears further discussion. This was the one indicator in which rural respondents perceived less improvement than their urban counterparts. The difficulties in providing home care through the COVID-19 pandemic were undoubtedly exacerbated in rural communities where there are fewer services. However, evidence consistently indicates that a home death may not be reasonable or preferable due to the resource and social contexts of rural communities [46]. Rather, it may be even more important to have safe places for death in rural communities than in urban communities where there are an abundance of home supports (e.g., meal/grocery delivery, home support, transportation services, equipment loan cupboards) [47]. If we seek to make significant gains in palliative care in Canada, attention to these in-community places to die for our rural and remote citizens must be high priority.

Findings from this survey are limited by the relatively small convenience sample that cannot be generalized to the larger population. However, this was a well-informed sample with the majority being healthcare professionals, of which 40% were working as specialized palliative care providers. Further, the majority of respondents had over 10 years of experience in palliative care. A second limitation was that data was collected during the COVID-19 pandemic and respondents’ perceived improvements, or lack thereof, were influenced by this context. As such, the data provides a picture of a system under strain which makes the perceived improvements even more significant. Repeating this survey in a post-pandemic context will be important. Also, further research is required to gain a better in-depth understanding of the improvements cited by these participants and how they can be leveraged further beyond the COVID pandemic.

## Conclusion

The Federal Governments’ Framework and Action Plan sets out a clear roadmap for improving palliative care in Canada. Even amidst the challenges of the COVID-19 pandemic, respondents to this survey perceived that notable improvement had been made in the areas of healthcare provider education, advance care planning, and the use of technology. It may be that the innovations required of the pandemic, such as greater use of technology, contributed to improvements that may not have occurred otherwise. However, they also indicated that aspects of support for family caregivers had worsened. Rural areas in Canada reported more significant improvements in the education, access, and research and data collection than their urban counterparts. Further leveraging these improvements will make an important contribution to solving some of the rural and remote palliative care issues that have arisen as a result of Canada’s unique geography.

## Data Availability

The datasets used and/or analyzed during the current study are available from the corresponding author on reasonable request.

## Abbreviation

WHO: World health organization
